# The heavy chain of 4F2 antigen promote prostate cancer progression via SKP-2

**DOI:** 10.1038/s41598-021-90748-9

**Published:** 2021-06-01

**Authors:** Maihulan Maimaiti, Shinichi Sakamoto, Masahiro Sugiura, Manato Kanesaka, Ayumi Fujimoto, Keisuke Matsusaka, Minhui Xu, Keisuke Ando, Shinpei Saito, Ken Wakai, Yusuke Imamura, Keiichi Nakayama, Yoshikatsu Kanai, Atsushi Kaneda, Yuzuru Ikehara, Jun-Ichiro Ikeda, Naohiko Anzai, Tomohiko Ichikawa

**Affiliations:** 1 Department of Urology, Chiba University Graduate School of Medicine, 1-8-1 Inohana, Chuo-ku, Chiba City, Chiba 260-8670 Japan; 2 Department of Tumor Pathology, Chiba University Graduate School of Medicine, Chiba, Japan; 3 Department of Molecular Oncology, Chiba University Graduate School of Medicine, Chiba, Japan; 4 Department of Pathology, Chiba University Hospital, Chiba, Japan; 5 Bio-System Pharmacology, Osaka University Graduate School of Medicine, Osaka, Japan; 6 Department of Pharmacology, Chiba University Graduate School of Medicine, Chiba, Japan; 7 Department of Molecular and Cellular Biology, Medical Institute of Bioregulation, Kyushu University, Fukuoka, Japan; 8 Department of Diagnostic Pathology, Graduate School of Medicine, Chiba University, Chiba, Japan

**Keywords:** Cancer, Cell biology, Molecular biology, Oncology, Urology

## Abstract

The 4F2 cell-surface antigen heavy chain (4F2hc) forms a heterodimeric complex with L-type amino acid transporter 1 (LAT1) and transports large neutral essential amino acids. However, in contrast to the traditional role of LAT1 in various cancers, the role of 4F2hc has largely remained unknown. The role of 4F2hc in prostate cancer was studied. Treatment of C4-2 cells with si4F2hc was found to suppress cellular growth, migratory and invasive abilities, with this effect occurring through the cell cycle, with a significant decrease in S phase and a significant increase in G0/G1 phase, suggesting cell cycle arrest. In addition, it was proven by RNA seq that the key to 4F2hc’s impact on cancer is SKP2. si4F2hc upregulates the protein expression of cyclin-dependent kinase inhibitors (P21cip1, P27kip1) through the downstream target SKP2. Furthermore, the expression of 4F2hc and LAT1 in prostate cancer cells suggests the importance of 4F2hc. Multivariate analysis showed that high 4F2hc expression was an independent prognostic factor for progression-free survival (HR 11.54, *p* = 0.0357). High 4F2hc was related to the clinical tumour stage (*p* = 0.0255) and Gleason score (*p* = 0.0035). Collectively, 4F2hc contributed significantly to prostate cancer (PC) progression. 4F2hc may be a novel marker and therapeutic target in PC.

## Introduction

Prostate cancer (PC) is the most commonly diagnosed malignancy and one of the most frequently diagnosed cancers in men^1,2^. Despite progress in the treatment of localized PC, management of locally advanced and metastatic disease is still a critical unmet need^3,4^. Androgen deprivation therapy (ADT) is the standard treatment for advanced PC^2^. However, the clinical benefit of ADT is only temporary, and it has always evolved into castration-resistant prostate cancer (CRPC) with different treatments^5,6^. A new generation of androgen receptor (AR)-targeting agents has been developed, such as the androgen biosynthesis inhibitor abiraterone and the AR inhibitor enzalutamide, and they have been shown to improve overall survival (OS)^6,7^. However, treatment resistance remains a significant challenge for CRPC. Some reports showed major improvement with abiraterone and enzalutamide treatment, whereas there was no decrease in serum prostate-specific antigen (PSA) levels^8,9^. Thus, a novel no-AR therapeutic approach and biomarker candidates for CRPC remain a significant issue.

Cancer cells require substantial amounts of nutrients to grow and multiply, especially cancer cells that have lost normal reproductive function. Amino acids are essential nutrients that are transported to the cell by a selective transporter on the plasma membrane^10–12^. Amino acid transporters are required for tumour growth and proliferation. Several lines of evidence have shown that nutrient transporters are upregulated in cancer cells while supporting large-scale growth and reproduction^13^.

L-type amino acid transporter 1 (LAT1, SLC7A5), a systemic L amino acid transporter, requires a covalent association with 4F2 cell-surface antigen heavy chain (4F2hc,SLC3A2) for its functional expression in the plasma membrane^14^. LAT1, which has been shown to bind to 4F2hc, is abundantly expressed in various types of cancers, including non-small cell lung cancer, breast cancer, biliary tract cancer, pancreatic cancer, and prostate cancer^15–19^. Besides LAT1, several studies have shown an association between higher 4F2hc expression levels and worse prognosis in various types of cancer^20–22^. In addition, we recently identified 4F2hc as one of the main target genes for AR-V7. Upregulation of 4F2hc through AR-V7 contributes to the progression of CRPC^23^.

Based on the above-described findings, it may be reasonable to expect that 4F2hc is a promising prognostic biomarker, as well as a molecular target, in PC. In this study, the oncogenic function of 4F2hc in PC and its relationship with the clinical outcome of PC patients was studied.

## Results

### Analysis of 4F2hc expression and 4F2hc knockdown in PC cell lines

**Figure 1:**
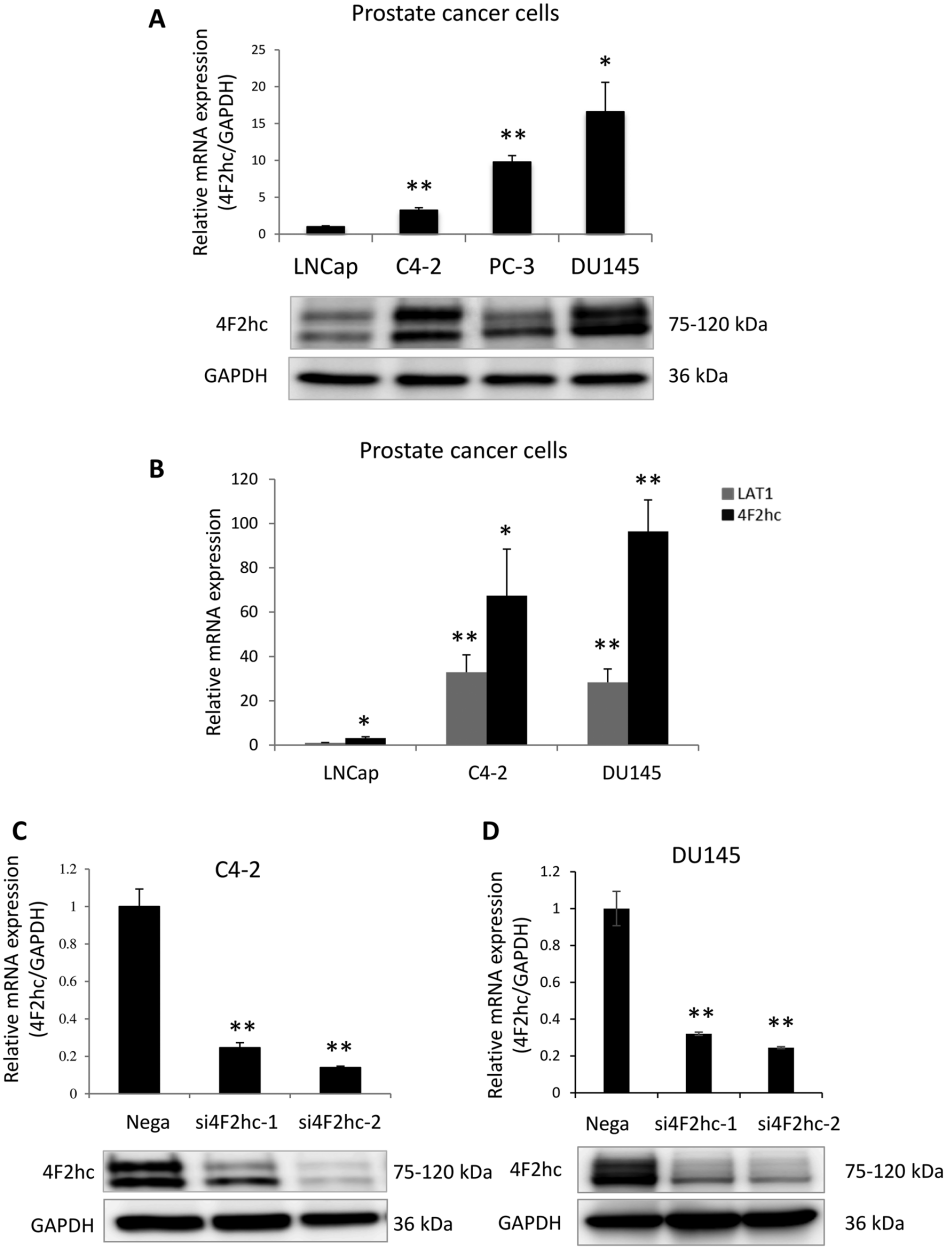
4F2hc signalling pathway. Cell cycle analysis was performed with si4F2hc (si4F2hc-1 and si4F2hc-2) and compared with a control group (**A** to **D**). After 72 h or 96 h, si4F2hc (si4F2hc-1 and si4F2hc-2) downregulation of SKP2, phosphorylation of AKT and MAPK, and increased expression of p21 and p27 (**E**). Data represent three independent experiments with similar results. Data for 4F2hc comes from the same experiment as depicted in Fig. 1C.

In PC cell lines, the highest 4F2hc protein expression was observed in DU145 cells, followed by PC-3 and C4-2 cells (Fig. 1A) (*p* = 0.0061, *p* = 0.0804, *p* = 0.0026, and *p* = 0.0200; respectively). Compared with LAT1 expression, significantly higher 4F2hc expression was observed in C4-2 and DU145 cells (Fig. 1B) (*p* = 0.0014 and *p* = 0.0020; respectively). According to the basal expression level in Fig. 1A, C 4-2 and DU145 cells were used for subsequent functional analysis. The expression levels of si4F2hc (si4F2hc-1 and si4F2hc-2) were significantly decreased in C4-2 and DU145 cells compared with those in the Negative Control (Nega) (Fig. 1C,E) (D: *p* = 0.0026 and *p* = 0.0043; D: *p* = 0.0034 and *p* = 0.0017; respectively).

**Figure 2:**
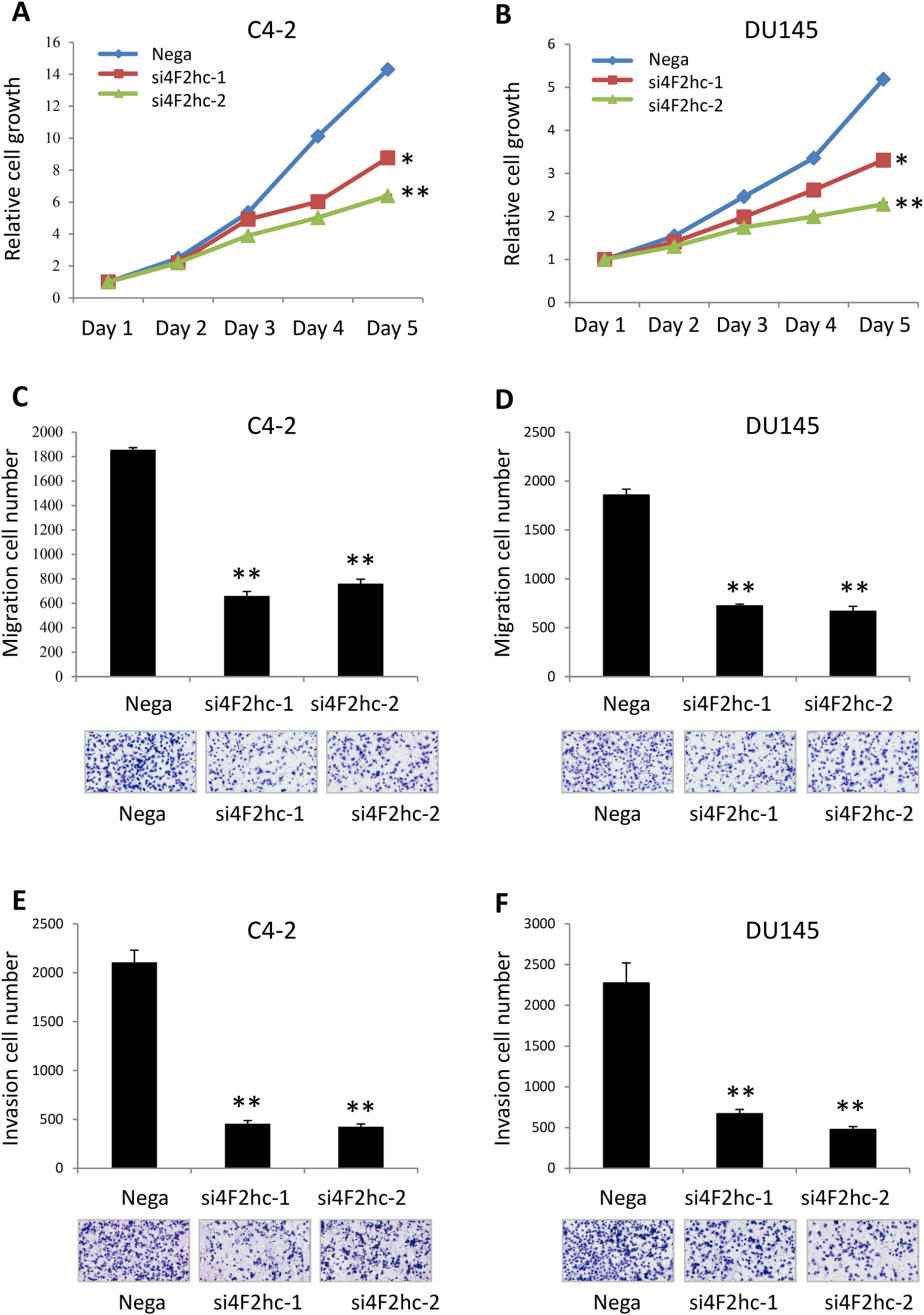
Functional significance of 4F2hc in DU145 and C4-2 cells. Si4F2hc (si4F2hc-1 and si4F2hc-2) inhibits C4-2 and DU145 cell proliferation (**A**,**B**), migration (**C**,**D**), and invasion (**E**,**F**). Nega indicates negative siRNA control. Data represent three independent experiments with similar results. P-values were calculated by the Mann–Whitney U-test. * p < 0.05, ** p < 0.01.

### The functional role of 4F2hc in PC

#### Si4F2hc suppresses cell proliferation, migration, and invasion

Levels of 4F2hc mRNA were significantly suppressed following transfection of C4-2 and DU145 cells with si4F2hc (si4F2hc-1 and si4F2hc-2) compared with mock-transfected cells or those transfected with Nega. The si4F2hc (si4F2hc-1 and si4F2hc-2)-transfected cells showed significant growth reduction compared to the Nega (Fig. 2A,B) (A: *p* = 0.0131 and *p* = 0.0012; B: *p* = 0.0177 and *p* = 0.0033; respectively). Furthermore, si4F2hc-transfected cells also demonstrated significant decreases in migration (Fig. 2C) (*p* = 0.0075 and *p* = 0.0003; D: *p* = 0.0011 and *p* = 0.0050; respectively) and invasion (Fig. 2E) (*p* = 0.0027 and *p* = 0.0015; F: *p* = 0.0079 and *p* = 0.0073; respectively) activities compared with Nega (Fig. 2C-F). Cell migration activity was evaluated with wound healing assays. (Fig. S1A) (*p* = 0.0031 and *p* = 0.0150; B: *p* = 0.0066 and *p* = 0.0087; respectively).

#### Analyse downstream genes by RNA-seq

**Figure 3:**
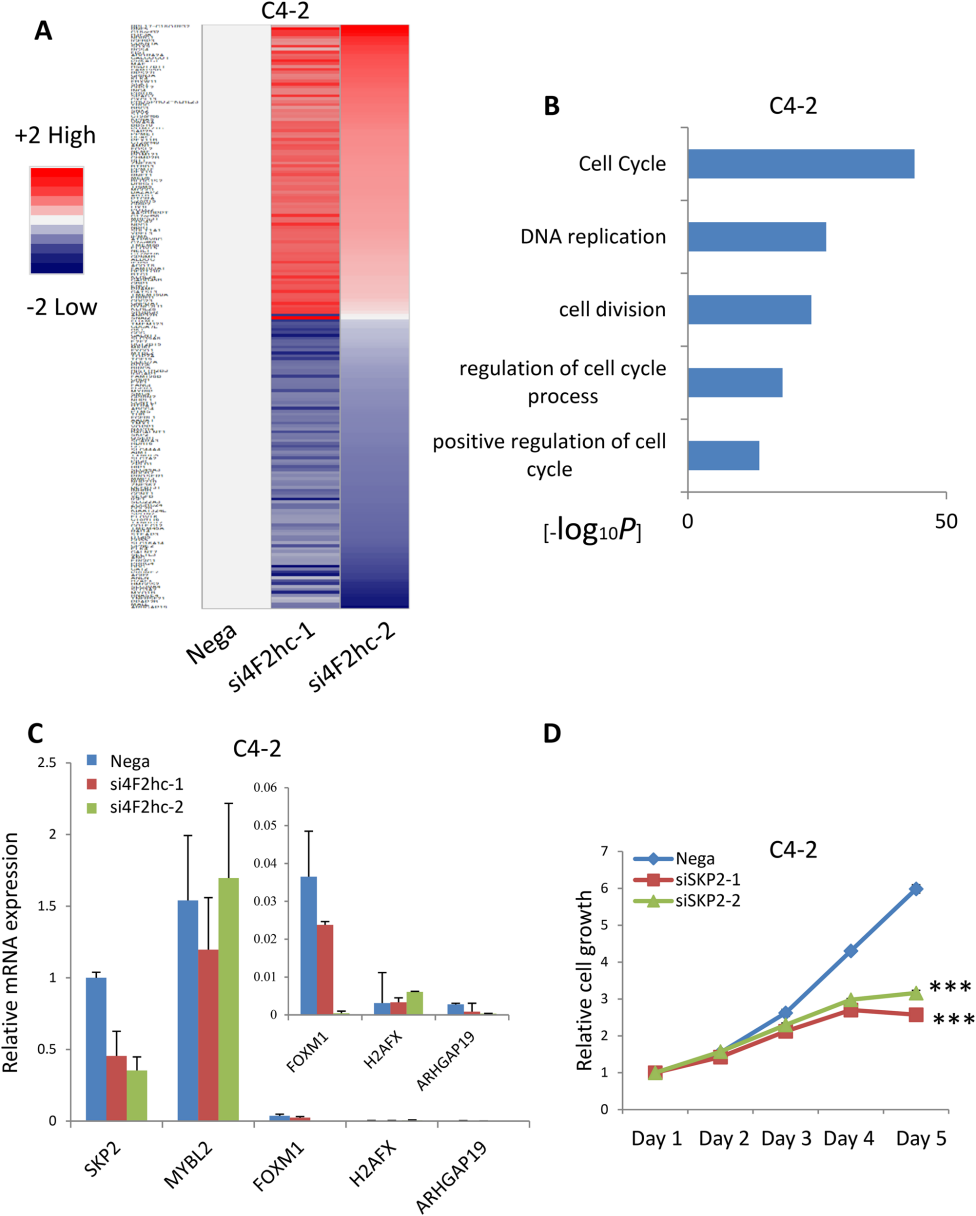
4F2hc RNA-sequencing analysis. RNA-seq analysis was performed with si4F2hc (si4F2hc-1 and si4F2hc-2) and compared with a control group (**A**). The most down-regulated 1403 genes are selected, and Matascape is used for analysis of significantly related with GO terms (**B**). Si4F2hc concentration-dependent effect on candidate genes was assessed by real-time PCR (**C**). SiSKP2 (siSKP2-1 and siSKP2-2) inhibits C4-2 cell proliferation (**D**). Data represent three independent experiments with similar results. P-values were calculated by the Mann–Whitney U-test. *** p < 0.001.

To further elucidate the mechanism underlying inhibition by si4F2hc, the gene target and downstream signals of si4F2hc were investigated. A comparative RNA-seq analysis was performed using si4F2hc (si4F2hc-1 and si4F2hc-2) and Nega. Figure 3A shows a heat map of changes in the RNA-seq analysis under the influence of si4F2hc. In order to make the heat map more concrete, the most down-regulated 1403 genes are selected, and Gene ontology (GO) analysis was performed using Metascape (http://www.metascape.org). These commonly downregulated genes were markedly associated with the cell cycle (−log10 = 43.84), DNA replication (-log10 = 26.75), and cell division (-log10 = 23.88) (Fig. 3B). Confirmation of target genes downregulated at the top of the cell cycle was performed by real-time PCR analysis. Finally, SKP2 was identified as a target gene of 4F2hc (Fig. 3C). To study the functional role of SKP2, the growth of siSKP2-transfected cells was monitored for five days. The increase in SiSKP2 (siSKP2-1 and siSKP2-2)-transfected cells was significantly reduced compared to Nega (Fig. 3D) (*p* = 0.0002 and *p* = 0.0005; respectively). We used C4-2 cells to evaluate RNA-seq results for identified genes (Fig. S2A). SiSKP2 (siSKP2-1 and siSKP2-2) inhibits DU145 cell proliferation (Fig. S2B) (*p* = 0.0076 and *p* = 0.0084; respectively).

In order to study the associations among 4Fhc, SKP2, and LAT1 expressions, the effects of si4F2hc on SKP2 and 4F2hc expressions and the effects of siSKP2 on 4F2hc and LAT1 expressions were studied. SKP2 and 4F2hc mRNA expressions were significantly lower in si4F2hc-transfected than in Nega (Fig. S3A to C) (A: *p* = 0.0011 and *p* = 0.0010; B: *p* = 0.0116 and *p* = 0.0021; C: *p* = 0.0155 and *p* = 0.0097; respectively). SKP2 mRNA expression was significantly lower in siSKP2-transfected than in Nega (Fig. S3, D) (*p* = 0.0019 and *p* = 0.0028; respectively). However, siSKP2 did not affect the expression of 4F2hc and LAT1 mRNA levels (Fig. S3E,F) (E: *p* = 0.1513 and *p* = 0.02484; D: *p* = 0.2362 and *p* = 0.6480; respectively).

Validation of the association between 4F2hc and SKP2 expression by rescue assay. The skp2 overexpression by plasmid did not change the 4F2hc expression (Fig. S4A) (*p* = 0.0011, *p* = 0.0031, *p* = 0.0017, *p* = 0.0027; respectively). But the SKP2 plasmid caused SKP2 overexpression in the 4F2hc knockout group (Fig. S4B) (*p* = 0.0085, *p* = 0.0026, *p* = 0.0036, *p* = 0.0226; respectively). 4F2hc plasmid allowed 4F2hc overexpression (Fig. S4C) (*p* = 0.2015, *p* = 0.1953, *p* = 0.0012, *p* = 0.0095; respectively). The 4F2hc plasmid gave a slightly increased expression of SKP2 (Fig. S4D) (*p* = 0.0005, *p* = 0.0004, *p* = 0.0008, *p* = 0.0004; respectively). The effect of the rescue assay was observed using cell proliferation. 4F2hc and SKP2 plasmids effectively accelerated cell proliferation (Fig. S4E,F).

#### Regulation of SKP2 and the cell cycle pathway by 4F2hc

**Figure 4:**
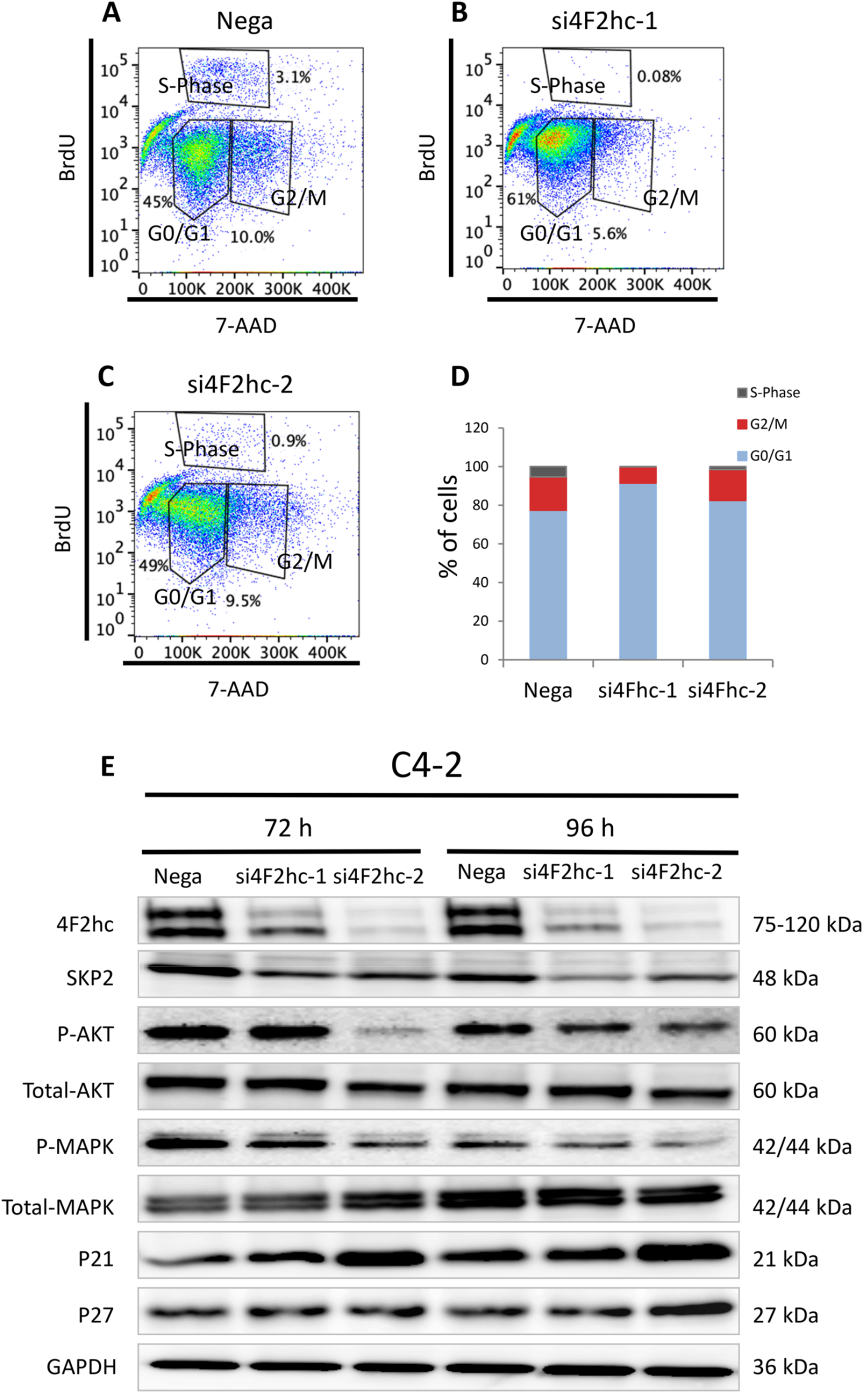
4F2hc signalling pathway. Cell cycle analysis was performed with si4F2hc (si4F2hc-1 and si4F2hc-2) and compared with a control group (**A** to **D**). After 72 h or 96 h, si4F2hc (si4F2hc-1 and si4F2hc-2) downregulation of SKP2, phosphorylation of AKT and MAPK, and increased expression of p21 and p27 (**E**). Data represent three independent experiments with similar results.

Given the key function of SKP2 in the cell cycle and its molecular interplay with P21cip1 and P27cip1, a potential role of SKP2 in cell cycle progression was investigated. Treatment of C4-2 cells with si4F2hc-1 led to a significant decrease in the S phase and a substantial increase in the G0/G1 phase, suggesting cell cycle arrest (Fig. 4A-C). The percentage of si4F2hc-transfected cells was higher in the G0/G1 phase than in Nega-transfected cells (Fig. 4D). Next, whether 4F2hc regulation is associated with the expression of cyclin SKP2 in these cells and the regulation of the expression level or activation state of downstream signals were investigated. Western blot analysis showed significant downregulation of SKP2 in a state in which 4F2hc was inhibited, inhibition of phosphorylation of MAPK and AKT, and upregulation of protein expression by cyclin-dependent kinase inhibitors (P21cip1, P27kip1) (Fig. 4E).

#### 4F2hc and LAT1 expression in PC tissue and association with clinical variables

**Figure 5:**
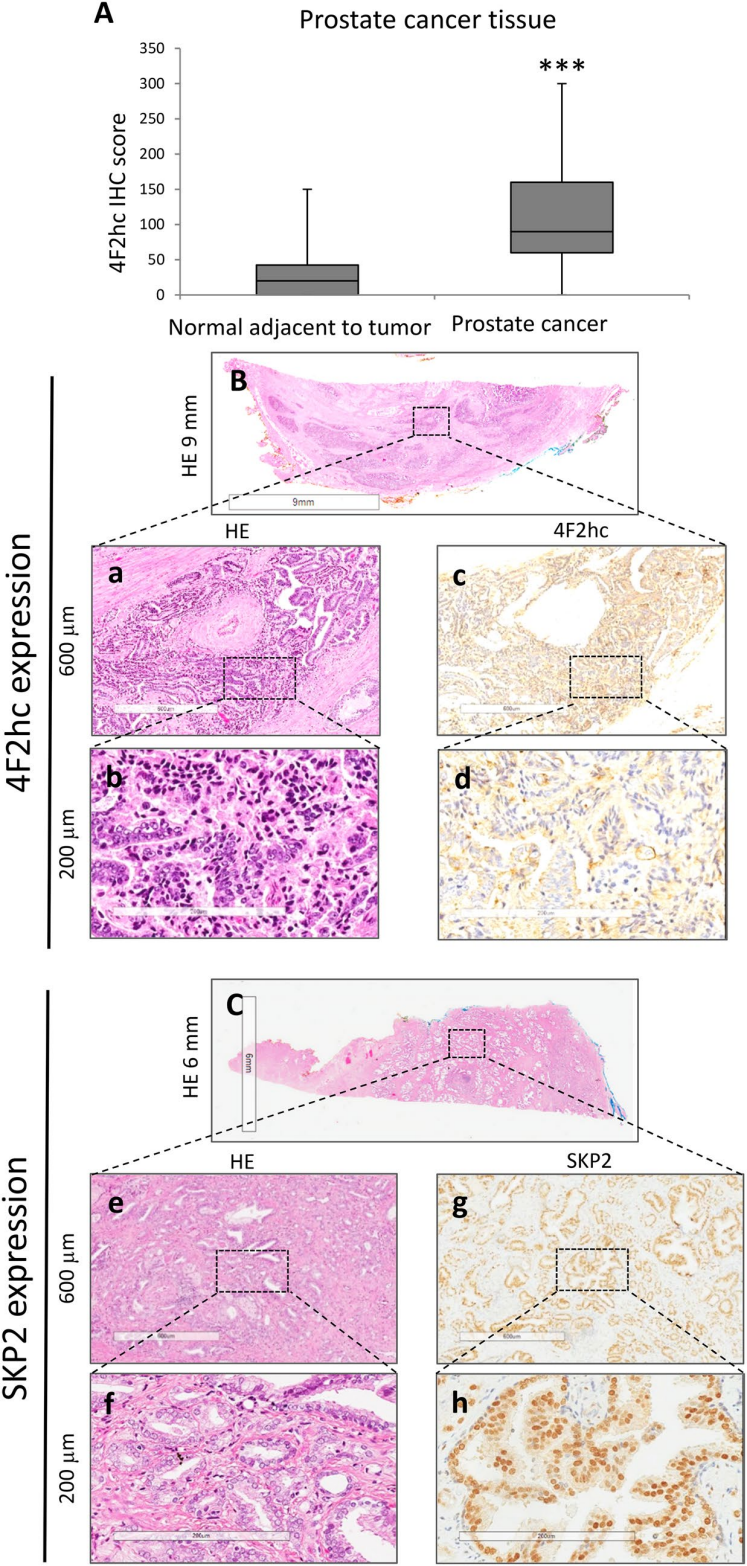
Representative staining pictures showing 4F2hc and SKP2 expression in PC tissues. Quantification of 4F2hc staining of NAT and PC tissues. The 4F2hc IHC scores levels are shown in normal prostate tissues and PC (**A**). 4F2hc and SKP2 expression in PC tissues was analysed by immunohistochemistry. Sections were stained with haematoxylin and eosin (**B**; a: 600 µm and b: 200 µm and **C**; e: 600 µm and f: 200 µm). Representative images of 4F2hc immunohistochemical expression (c: 600 µm and d: 200 µm) and representative images of SKP2 immunohistochemical expression (g: 600 µm and h: 200 µm). Representative staining images are shown.

The clinical significance of 4F2hc was further investigated, along with examination of 4F2hc expression in PC specimens, by IHC. Positive immunostaining for 4F2hc was detected in the cell membrane and cytoplasm. Intense immunostaining for 4F2hc was detected in cancerous lesions, whereas non-malignant lesions showed negative or weak immunostaining. Positive immunostaining for SKP2 was detected in the nucleus, mainly on the superficial central compartment of the tumour rather than in the tumour margins. The expression level of 4F2hc was studied by IHC. Positive immunostaining for 4F2hc was detected in the cell membrane and cytoplasm by PC specimens IHC. A strong 4F2hc immune response was detected in the cancer lesion`s cell membrane, whereas normal adjacent tumour (NAT) mainly showed weak immunostaining. Regarding the 4F2hcIHC scores in NAT and PC lesions, NAT ranged from 0 to 150 (median = 20), whereas PC lesions ranged from 0 to 300 (median = 90). The 4F2hc IHC score was significantly higher in PC lesion than in NAT (Fig. 5A; *p* = 0.0001). Figure 5B,C are representative IHC result for 4F2hc and SKP2 staining. Sections were stained with haematoxylin and eosin (Fig. 5B; a: 600 µm; b: 200 µm; and C; e: 600 µm; f: 200 µm), and representative images of 4F2hc IHC expression (Fig. 5C: 600 µm; d: 200 µm) and SKP2 IHC expression (Fig. 5G: 600 µm; h: 200 µm) are shown. LAT1 IHC is shown in Fig. S5. Sections were stained with haematoxylin and eosin (Fig. S5A: a: 600 µm; b: 200 µm), and representative images of LAT1 IHC expression are shown (Fig. S5c: 600 µm; d: 200 µm).

**Figure 6:**
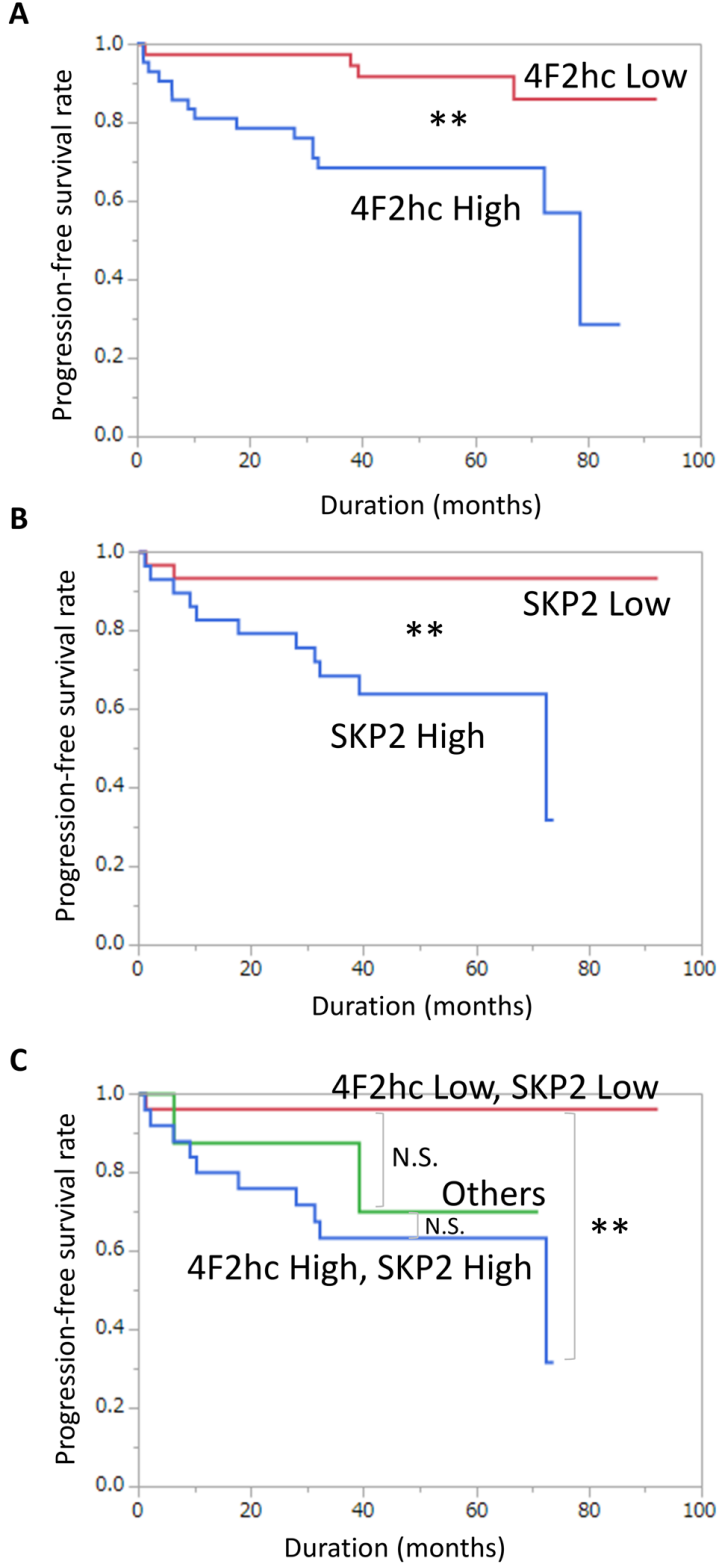
Progression-free survival of PC patients categorized by 4F2hc and SKP2 expression. Prognostic significance of 4F2hc expression for progression-free survival (PFS) (**A**), and prognostic significance of SKP2 expression for (PFS) (**B**). Prognostic significance of high 4F2hc/high SKP2 expression and low 4F2hc/low SKP2 expression, others are low 4F2hc/high SKP2 or high 4F2hc/low SKP2 (**C**). N.S. No significant difference. ** p < 0.01.

Next, the prognostic clinical significance of 4F2hc and LAT1 expression was evaluated statistically. The patients’ characteristics are listed in Table 1. Specimens were divided into two groups based on median 4F2hc and LAT1 IHC scores. The results showed that the high-4F2hc expression group showed significantly shorter progression-free survival (PFS) than the low 4F2hc expression group (Fig. 6A; *p* = 0.0034). Although the high LAT1 expression group tended to show shorter survival, LAT1 expression was not related to PFS (Fig. S6A; *p* = 0.1085). When combining 4F2hc and LAT1 expression, the high 4F2hc/high LAT1 expression group showed the worst PFS (*p* = 0.0071), followed by the high 4F2hc/low LAT1 or low 4F2hc/high LAT1 expression group (others) (*p* = 0.2297). In contrast, the low 4F2hc/low LAT1 expression group showed good PFS (Fig. S6B).

**Table 1:** Patients’ characteristics.

Clinical factor (n = 82)	Median (range) or n (%)
Age (y)	66 (50–75)
cT stage (n)	
1	54 (69.23%)
2	13 (16.67%)
3	11 (14.10%)
GS (n)	
6	24 (29.27%)
7	37 (45.12%)
8	12 (14.63%)
9	9 (10.98%)
TST (ng/dL)	4.64 (0.42–10.9)
PSA (ng/mL)	7.87 (2.45–31.78)
PSAD	0.30 (0.06–1.44)

cT stage = clinical tumour stage, GS = Gleason score, TST = testosterone, PSA = prostate-specific antigen, PSAD = PSA density.

Next, associations between clinicopathological characteristics and 4F2hc protein expression were investigated. On multivariate analysis, high 4F2hc expression (HR 11.54, *p* = 0.0357) and high clinical tumour stage (CT stage) (HR 4.22, *p* = 0.0280) were also identified as independent prognostic factors for PFS (Table 2). The relationships between 4F2hc expression and various clinical factors were determined. High 4F2hc expression was associated with high age (*p* = 0.0162), high cT stage (*p* = 0.0255), and high Gleason score (GS) (*p* = 0.0035) in PC patients (Table 3). However, high LAT1 expression was only associated with high GS (*p* = 0.0399) in PC patients (Table S1).

**Table 2:** Predictors of progression-free survival.

	Univariate analysis				Multivariate analysis			
	Cut off	HR	95% CI	*P*	HR	95% CI	*P*	
**Age (y)**	66	0.96	0.37–2.43	0.9281				
**cT stage**	2	4.86	1.84–14.16	0.0014	4.22	1.64–18.73	0.0280	*
**GS**	7	3.06	1.00–13.25	0.0488	1.78	0.24–36.35	0.6028	
**TST (ng/dL)**	4.64	0.69	0.26–1.76	0.4364				
**PSA (ng/mL)**	7.90	1.74	0.70–4.51	0.2284				
**4F2hc Score**	High/Low	4.59	1.65–16.25	0.0027	11.54	1.16–276.67	0.0357	*
**LAT1 Score**	High/Low	2.68	0.87–11.64	0.0886				
**SKP2 Score**	High/Low	6.92	1.83–45.07	0.0029	0.73	0.14–6.99	0.7534	

HR = Cox proportional hazard ratio, 95% CI = 95% confidence interval, cT stage = clinical tumour stage, GS = Gleason score, TST = testosterone, PSA = prostate-specific antigen.

*Statistical significance (*p* < 0.05).

**Table 3:** Comparison of clinical factors between 4F2hc Low and 4F2hc High groups.

	4F2hc Low	4F2hc High	P	
Age (y)	64.50 ± 5.25	66.50 ± 5.20	0.0162	*
cT stage (n)			0.0255	*
1	29	25		
2	5	8		
3	2	9		
GS (n)			0.0035	*
6	17	7		
7	19	18		
8	3	9		
9	1	8		
TST (ng/dL)	4.66 ± 1.92	4.64 ± 1.78	0.1661	
PSA (ng/mL)	7.46 ± 6.37	8.30 ± 6.57	0.3781	
PSAD	0.27 ± 0.23	0.30 ± 0.31	0.1156	

Data are expressed as means ± standard deviation unless otherwise indicated. cT stage = clinical tumour stage, GS = Gleason score, TST = testosterone, PSA = prostate-specific antigen, PSAD = PSA density.

*Statistical significance (*p* < 0.05).

#### 4F2hc and SKP2 expression in PC tissue and associations with clinical variables

The prognostic clinical significance of 4F2hc and SKP2 expressions was evaluated statistically. The high SKP2 expression group also showed a significantly shorter PFS (Fig. 6B; *p* = 0.0040). When expressed in conjunction with 4F2hc and SKP2, the high 4F2hc/SKP2 high expression group showed the worst PFS (*p* = 0.0017), followed by the high 4F2hc/low SKP2 or low 4F2hc/high SKP2 expression group (others) (*p* = 0.0623). In contrast, the low 4F2hc/low LAT1 expression group showed good PFS (Fig. 6C). The relationship between SKP2 expression and various clinical factors was determined. High SKP2 expression was associated with high cT stage (*p* = 0.0277), high GS (*p* = 0.0138), high PSA (*p* = 0.0458), and high PSAD (*p* = 0.0360) in PC patients (Table 4).

**Table 4:** Comparison of clinical factors between SKP2 Low and SKP2 High groups.

	SKP2 Low	SKP2 High	P	
Age (y)	64.23 ± 5.47	66.57 ± 5.33	0.0998	
cT stage (n)			0.0277	*
1	25	16		
2	2	8		
3	2	5		
GS (n)			0.0138	*
6	13	6		
7	14	13		
8	1	6		
9	2	5		
TST (ng/dL)	5.38 ± 1.87	4.51 ± 1.74	0.0984	
PSA (ng/mL)	8.68 ± 4.78	12.06 ± 7.70	0.0458	*
PSAD	0.30 ± 0.19	0.43 ± 0.29	0.0360	*

Data are expressed as means ± standard deviation unless otherwise indicated. cT stage = clinical tumour stage, GS = Gleason score, TST = testosterone, PSA = prostate-specific antigen, PSAD = PSA density.

*Statistical significance (*p* < 0.05).

To verify the reliability of our data, statistical analysis was performed using the Cancer Genome Atlas (TCGA) data. TCGA data and our data showed the similar results. The results showed that the High 4F2hc expression group also showed a significantly shorter PFS (*p* = 0.0369) (Fig. S7A). Similarly, the high-SKP2 expression group showed significantly shorter PFS than the low SKP2 expression group(*p* = 0.0088) (Fig. S7B), but LAT1 expression was not related to PFS (*p* = 0.2166) (Fig. S7C). When combining 4F2hc and SKP2 expression, the low 4F2hc/low SKP2 expression group showed good PFS. In contrast, the high 4F2hc/high LAT1 expression group showed the worst PFS (*p* = 0.0006) (Fig. S7D). When combining 4F2hc and LAT1 expression, the low 4F2hc/low LAT1 expression group showed good PFS (*p* = 0.0273) (Fig. S7E).

## Discussion

The present study demonstrated several novel findings. 4F2hc expression seems to be one of the promising therapeutic targets in PC. Of the major clinical factors, including LAT1 expression, 4F2hc expression was the most significant prognostic factor in PC patients. The inhibition of 4F2hc function prevents the progression of several PC cell types. Furthermore, SKP2 was newly identified as a novel target of 4F2hc. Current evidence indicated the contribution of the cell cycle pathway as a central downstream mechanism of 4F2hc^22,24^.

4F2hc expression is increased in various human neoplasms, such as gastric cancer, pulmonary pleomorphic carcinoma, and neuroendocrine carcinoma^20–22^. Moreover, it has been reported that increased 4F2hc expression is significantly associated with shorter survival outcomes, cell proliferation, and metastasis^25^. 4F2hc binds with LAT1 on the membranous surface of cancer cells^26^. After the discovery of LAT1, it was also shown that the other five members of the solute carrier family 7 bind to 4F2hc as a light chain to form a different heterodimeric amino acid transporter. Heterodimerization is essential for its functional expression^27^. Interestingly, relevant research shows that ATF4 regulates 4F2hc, LAT1, and ASCT1 gene, with low expression in healthy prostate tissue and early prostate cancer. However, they are significantly increased in metastatic prostate cancer, indicating the essential nutrients required for transporters to promote metastatic prostate cancer^28^.

4F2hc antigen is a heterodimeric protein composed of a glycosylated heavy chain (120 kDa) and a non-glycosylated light chain (75 kDa)^14,29^. The light and heavy chains were found to have unique biochemical functions. The heavy chain of CD98 is associated with functional integrin 4 and can regulate integrin activation through early T-cell antigen activation function^30^. Binding of 4F2hc to β1 integrin has been reported to be specific. primarily by promoting cell spreading and tumorigenicity, a process that is mediated by stimulation of β1-dependent adhesion^31^. Because it is involved in cell growth, cell adhesion and other cellular activities. The light chain exhibits a function mainly in amino acid transport^32^. In addition to leucine import via amino acid transporter proteins LAT-1 and LAT-2, the light chain can also bind to xCT, y^+^ LAT1, y^+^ LAT2 and asc1^33,34^. Heterodimeric substrates differ in the survival and growth of many cell types. Importantly, the light chain cannot be expressed independently, the presence of the heavy chain is essential, and the isolated heavy chain can function independently in amino acid transport^35^. This specificity is shown in various cancers, such as: renal cancer^27^, Non-Small Cell Lung cancer^36^ and oral cancer^37^.

In previous studies, the oncogenic function of 4F2hc was demonstrated. Several groups reported that 4F2hc inhibits the proliferation of cancer cells by inhibiting the cell cycle. The FACS assay was performed in C4-2 cells. Si4F2hc effectively inhibited the S phase, with a significant increase in the G0/G1 period, suggesting cell cycle arrest. These data are consistent with two previous studies reporting the effect of 4F2hc on human osteosarcoma and thymic epithelial tumors^24,38^. 4F2hc has been shown to affect cancer cell proliferation through the AKT, MAPK, and cell cycle related P21 and P27 signal pathways.

SKP2 is related to a cell cycle signal pathway. It is worth noting that overexpression of SKP2 is associated with the promotion and aggravation of many tumors^39,40^. The cell cycle signal pathway is activated in PC. Therefore, inhibition of SKP2 promotes the deterioration of cancer by targeting promotion of p21 and p27 and by cell senescence^41^. Reports show that overexpression of SkP2 coupled with under-expression of p27 is characteristic of CRPC^42^. In line with this evidence, the present data implicated the participation of SKP2 through alteration of the S phase of the cell cycle in the 4F2hc-related pathway. Previous studies demonstrated that mucin 1, mucin 16, and mucin 5B were the downstream genes of 4F2hc in gastric carcinoma cells^43^, although the effect of 4F2hc on transcription remains mostly unknown. In the present study, a comprehensive whole transcriptome shotgun sequencing analysis was conducted using the C4-2 PC cell line and Metascape gene analysis. The data showed that downregulated genes were markedly associated with the cell cycle, DNA replication, and cell division, and identified SKP-2 as a specific target gene among the downregulated genes in C4-2 cells.

Nevertheless, there are several limitations in this study. First, the data were obtained from a single institution, and the number of patients and the follow-up periods were limited. A prospective, multi-institutional study would be ideal to objectively assess the prognostic significance of 4F2hc. Second, the mechanism of how 4F2hc regulates SKP2 remained to be identified. We are currently performing a chip assay to verify any direct association between two genes. Third, although the major binding partner of 4F2hc may be LAT1, the functional relationships with other transporters need to be determined.

Collectively, the present results suggest that, in line with its high expression frequency, 4F2hc may be a promising prognostic marker for PC patients. Thus, it is possible that high levels of 4F2hc expression, together with a high level of LAT1 expression, in surgical specimens may indicate the need to follow-up the patient carefully with frequent imaging in order to take precautions against recurrence.

## Materials and methods

### PC tissue specimens

A total of 82 clinical PC tissue samples were obtained from patients who underwent radical prostatectomy at Chiba University Hospital between 2006 and 2014. The present study was conducted in accordance with ethical standards that promote and ensure respect and integrity for all human subjects and the Declaration of Helsinki. All experiments were performed in accordance with relevant named guidelines and regulations. The study was approved by the Chiba University Review Board (approval number 408), and the patients signed a written, informed consent form.

### Reagents and antibodies

In this study, siRNAs 4F2 cell-surface antigen heavy chain (si4F2hc) (Stealth siRNAs: HSS109825 and HSS109826), siRNAs S-Phase Kinase Associated Protein 2 (siSKP2) (Stealth siRNAs: HSS185711 and HSS109780), siRNA Negative Control (Stealth RNAi, Thermo Fisher Scientific, MA, USA), Lipofectamine 3000 Transfection Reagent (Invitrogen), were used. The antibodies used in the study were: anti-4F2hc (CD98, Santa Cruz Biotechnology, TX, USA); anti-4F2hc (CD98, Trans Genic,Tokyo, Japan); anti-LAT1 (Trans Genic); anti-SKP2, anti-MAPK, anti-phosphorylated MAPK, anti-AKT, anti-phosphorylated AKT, anti-p21cip1, and anti-p27kip1 (Cell Signaling Technology, Danvers, MA, USA); Anti-GAPDH (Ambion, Waltham, CA, USA).

### Cell culture and transfection

LNcap and C4-2 cell line were obtained from the American Type Culture Collection (Manassas, VA, USA). PC-3 cell line was obtained from the Cell Resource Canter for Biomedical Research, Institute of Development, Aging and Cancer Tohoku University (Sendai, Japan). DU145 cell line was obtained from the RIKEN Cell Bank (Tsukuba, Japan). PC cell lines were supplemented with 10% foetal bovine serum (FBS) in RPMI 1640 culture medium and maintained in a humidified atmosphere incubator (95% air, 5% CO_2_, 37 °C). Lipofectamine RNAiMAX reagent (Invitrogen) was used, and siRNA was transfected with PC cells. Detailed experimental methods have been previously reported^34^.

### mRNA expression evaluation

Total RNA was isolated using the RNeasy Mini Kit. cDNA was synthesized with the ImProm-II Reverse (Promega, Madison, WI, USA). mRNA expression was carried out as described previously^34^ with ABI 7300 (Applied Biosystems, Foster, CA, USA) and SYBR Green PCR Master Mix (QPS-201, Toyobo, Japan). GAPDH (internal control) and PCR primers used in this study are listed in Table S2.

### Western blot analysis

Protein expression levels were measured using GAPDH as the control. Protein samples (24 mg) were subjected to SDS-PAGE and transferred to Hybond-C membranes (GE Healthcare, Chicago, IL, USA). The membranes were then blocked (5% skim milk, 30 min, 37 °C). The primary antibody was incubated overnight at 4 °C. Detailed experimental methods have been previously reported^34^. Western blotting's uncropped data is in the supplementary information.

### Immunohistochemistry (IHC)

Immunohistochemistry procedures were performed according to a previously described method^34^. The slides were treated with endogenous peroxidase (30% hydrogen peroxide solution, 100% methanol, 10 min), and then incubated with anti-4F2hc and anti-SKP2 (4 °C overnight). Finally, the slides were lightly counterstained with haematoxylin, dehydrated with ethanol, cleared with xylene, and mounted.

IHC scores were as follows: 3 intense staining; 2 moderate; 1 very weak; and 0 no staining. IHC scores were calculated as follows: IHC score = 3 × (mean percentage of intensely stained cells in the field) + 2 × (mean percentage of moderately stained cells in the field) + 1 × (mean percentage of weakly stained cells in the field). Two independent investigators blinded to patient clinical status scored each specimen.

### Growth assay, cell migration, and invasion assay

Detailed experimental methods have been previously reported^34^. Following the manufacturers’ instructions, the migration assay was performed using the Cell Counting Kit-8 (343–07,623, Dojindo, Japan) and the Falcon Permeable Support Plate (353,097, Corning, NY, USA), and the invasion assay used the Matrigel invasion chamber (354,480, Corning). Cell migration and invasion assays were performed using the Diff-Quik kit (16,920, Funakoshi Co, Tokyo, Japan). Cell migration activity was also evaluated with wound healing assays. C4-2 and DU145 cells (2 × 10^5^) were plated in 6-well plates and incubated for 48 h with 10 µg/ml mitomycin C solution (20,898–21, Nacalai Tesque, Kyoto, Japan). Scraping cell monolayers with P-20 micropipette tips. The initial gap length (0 h) and the residual gap length (24 h) after wounding were calculated from the micrographs.

### Flow cytometric analysis

For cell cycle analysis, cells were stained with propidium iodide using the FITC BrdU Flow Kit (51-2359KC, BD Biosciences, Bedford, MA, USA) according to the manufacturer’s instructions and examined using the FACS Celesta Analyzer (660,344, BD Biosciences). Flow cytometry data were analysed using FlowJo using the previously reported flow cytometric method^34^.

### RNA sequencing

In order to examine the downstream signal of 4F2hc, RNA sequencing was performed based on previous reports^34^, using the SMART-Seq v4 Ultra Low Input RNA Kit (Clontech, Palo Alto, CA, USA), NEBNext Ultra DNA Library Prep Kit (New England Biolabs, Ipswich, MA, USA), HiSeq 1500 system (Illumina, Santiago, CA, USA), and Cuffdiff (Cufflinks version 2.2.1).

### Statistical analysis

The Kaplan–Meier method and univariate and multivariate Cox proportional models were used for statistical analyses. Mathematical calculations were performed using JMP Pro 15 (SAS Institute, Cary, NC, USA). The Cancer Genome Atlas (TCGA) (https://www.cancer.gov/) was obtained through Oncomine (https://www.oncomine.org).

## Supplementary Information

Supplementary Information.
