# Author Correction: The heavy chain of 4F2 antigen promote prostate cancer progression via SKP-2

**DOI:** 10.1038/s41598-025-89150-6

**Published:** 2025-02-21

**Authors:** Maihulan Maimaiti, Shinichi Sakamoto, Masahiro Sugiura, Manato Kanesaka, Ayumi Fujimoto, Keisuke Matsusaka, Minhui Xu, Keisuke Ando, Shinpei Saito, Ken Wakai, Yusuke Imamura, Keiichi Nakayama, Yoshikatsu Kanai, Atsushi Kaneda, Yuzuru Ikehara, Jun‑Ichiro Ikeda, Naohiko Anzai, Tomohiko Ichikawa

**Affiliations:** 1 Department of Urology, Chiba University Graduate School of Medicine, 1‑8‑1 Inohana, Chuo‑ku, Chiba City, Chiba 260‑8670 Japan; 2 Department of Tumor Pathology, Chiba University Graduate School of Medicine, Chiba, Japan; 3 Department of Molecular Oncology, Chiba University Graduate School of Medicine, Chiba, Japan; 4 Department of Pathology, Chiba University Hospital, Chiba, Japan; 5 Bio‑System Pharmacology, Osaka University Graduate School of Medicine, Osaka, Japan; 6 Department of Pharmacology, Chiba University Graduate School of Medicine, Chiba, Japan; 7 Department of Molecular and Cellular Biology, Medical Institute of Bioregulation, Kyushu University, Fukuoka, Japan; 8 Department of Diagnostic Pathology, Graduate School of Medicine, Chiba University, Chiba, Japan

Correction to: *Scientific Reports* 10.1038/s41598-021-90748-9, published online 5 March 2021

The original version of this Article contained errors.

As a result of an error during figure assembly, the Article contained a duplication of Fig. 1C 4F2hc in Fig. 1D 4F2hc. Since the original data for Fig. 1D was not available the Authors repeated the experiments, and Fig. 1D is now corrected with this new data, and the original images in SI file are also now updated. Additionally, Fig. 1C 4F2hc is duplicated in Fig. 4E 4F2hc. This duplication is intentional, as it represents the same experiment, but should have been disclosed.

Consequently, the legend of Fig. 1 was corrected as follows:

“4F2hc signalling pathway. Cell cycle analysis was performed with si4F2hc (si4F2hc-1 and si4F2hc-2) and compared with a control group (A to D). After 72 h or 96 h, si4F2hc (si4F2hc-1 and si4F2hc-2) downregulation of SKP2, phosphorylation of AKT and MAPK, and increased expression of p21 and p27 (E). Data represent three independent experiments with similar results.”

now reads:

“4F2hc signalling pathway. Cell cycle analysis was performed with si4F2hc (si4F2hc-1 and si4F2hc-2) and compared with a control group (A to D). After 72 h or 96 h, si4F2hc (si4F2hc-1 and si4F2hc-2) downregulation of SKP2, phosphorylation of AKT and MAPK, and increased expression of p21 and p27 (E). Data represent three independent experiments with similar results. Data for 4F2hc comes from the same experiment as depicted in Fig 1C.”

The original Fig. 1 and accompanying legend appear below.

**Fig. 1:**
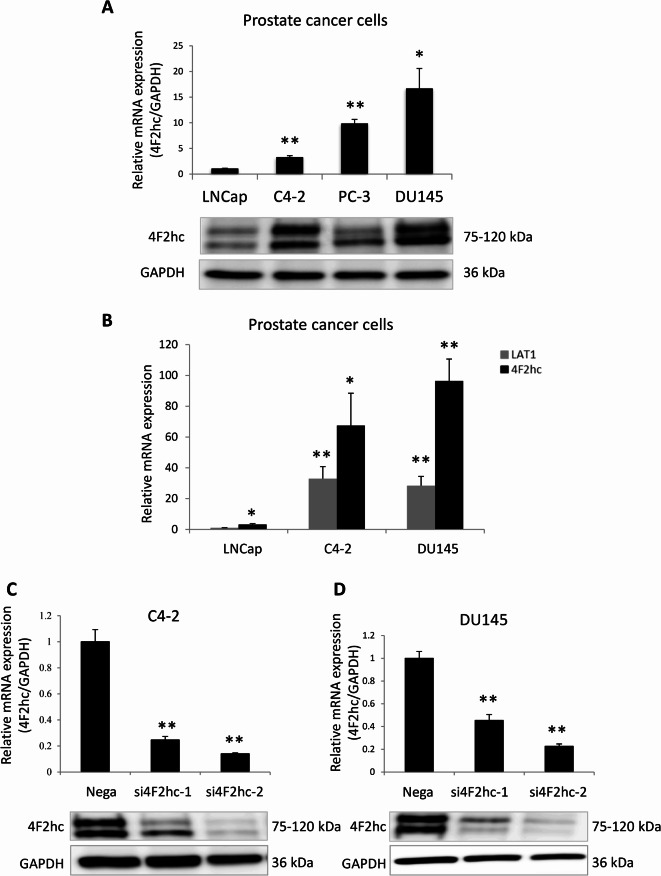
4F2hc signalling pathway. Cell cycle analysis was performed with si4F2hc (si4F2hc-1 and si4F2hc-2) and compared with a control group (**A**–**D**). After 72 h or 96 h, si4F2hc (si4F2hc-1 and si4F2hc-2) downregulation of SKP2, phosphorylation of AKT and MAPK, and increased expression of p21 and p27 (**E**). Data represent three independent experiments with similar results. Data for 4F2hc comes from the same experiment as depicted in (**C**).

The original Article has been corrected.

