# Supplementary material for: The heavy chain of 4F2 antigen promote prostate cancer progression via SKP-2
Source: Sci Rep. 2021 Jun 1;11:11478. doi: 10.1038/s41598-021-90748-9 (PMC8169706; doi:10.1038/s41598-021-90748-9)

The heavy chain of 4F2 antigen promote prostate cancer progression via SKP-2

Maihulan Maimaiti[1,2], †Shinichi Sakamoto[1*], Masahiro Sugiura[1,3], Manato Kanesaka[1,3], Ayumi Fujimoto[1], Keisuke Matsusaka[4], Minhui Xu[5], Keisuke Ando[1,6], Shinpei Saito[1,6], Ken Wakai[1,2], Yusuke Imamura[1], Keiichi Nakayama[7], Yoshikatsu Kanai[5], Atsushi Kaneda[3], Yuzuru Ikehara[2], Jun-Ichiro Ikeda[8], Naohiko Anzai[6], Tomohiko Ichikawa[1]

[1] *Department of Urology, Chiba University Graduate School of Medicine, Chiba, Japan*

[2] *Department of Tumor Pathology, Chiba University Graduate School of Medicine, Chiba, Japan*

[3] *Department of Molecular Oncology, Chiba University Graduate School of Medicine, Chiba, Japan*

[4] *Department of Pathology, Chiba University Hospital, Chiba, Japan.*

[5] *Bio-system Pharmacology, Osaka University Graduate School of Medicine, Osaka, Japan*

[6] *Department of Pharmacology, Chiba University Graduate School of Medicine, Chiba, Japan*

[7]*Department of Molecular and Cellular Biology, Medical Institute of Bioregulation, Kyushu University*

[8]*Department of Diagnostic Pathology, Graduate School of Medicine, Chiba University, Chiba, Japan.*

[*]Corresponding author: Shinichi Sakamoto
Chiba University Graduate School of Medicine
1-8-1 Inohana, Chuo-ku, Chiba City, Chiba 260-8670, Japan
Tel: +81-43-226-2134; Fax: +81-43-226-2136
Email: rbatbat1@gmail.com
†Equal study contribution

Table S1. Comparison of clinical factors between LAT1 Low and LAT1 High groups

	LAT1 Low	LAT1 High	P	
**Age (y)**	66.00 ± 5.39	65.50 ± 5.32	0.5578	
**cT stage (n)**			0.3108	
**1**	17	36		
**2**	2	11		
**3**	3	8		
**GS (n)**			0.0399	*
**6**	11	13		
**7**	7	29		
**8**	4	8		
**9**	3	9		
**TST (ng/dL)**	5.49 ± 2.16	4.61 ± 1.72	0.0928	
**PSA (ng/mL)**	10.07 ± 6.79	7.53 ± 6.40	0.7046	
**PSAD**	0.27 ± 0.26	0.30 ± 0.29	0.4738	

Data are expressed as means ± standard deviation unless otherwise indicated. cT stage = clinical tumour stage, GS = Gleason score, TST = testosterone, PSA = prostate-specific antigen, PSAD = PSA density

Table S2. Primer sequences for real-time PCR

Gene	Primer Type	Sequence (5′ to 3′)
4F2hc	**Forward**	ACCCCTGTTTTCAGCTACGG
	**Reverse**	GGTCTTCACTCTGGCCCTTC
LAT1	**Forward**	AGGAGCCTTCCTTTCTCCTG
	**Reverse**	CTGCAAACCCTAAGGCAGAG
SKP2	**Forward**	GATGTGACTGGTCGGTTGCTGT
	**Reverse**	GAGTTCGATAGGTCCATGTGCTG
MYBL2	**Forward**	AAAACAGTGAGGAGGAAC
	**Reverse**	CAGGGAGGTCAAATTTAC
FOXM1	**Forward**	GCATGAGGATGATTCTGACCT
	**Reverse**	GATCTGTGGACCACAAGATC
H2AFX	**Forward**	GGGCCTAGCTATCCCTCTCCCT
	**Reverse**	CTGCAAAAGTTCCAGTTCAGAAGCCAGA
ARHGAP19	**Forward**	AGATCTATGGCGACTGAGGCACAGAG
	**Reverse**	GAATTCTGCATGGACCATAGGAGACA

Figure S1. Cell migration activity was evaluated with wound healing assays (A and B). Scraping cell monolayers with P-20 micropipette tips. The initial gap length (0 h) and the residual gap length (24 h) after wounding were calculated from the micrographs. Data represent three independent experiments with similar results. P-values were calculated by the Mann–Whitney U-test. *P<0.05, **P<0.01.

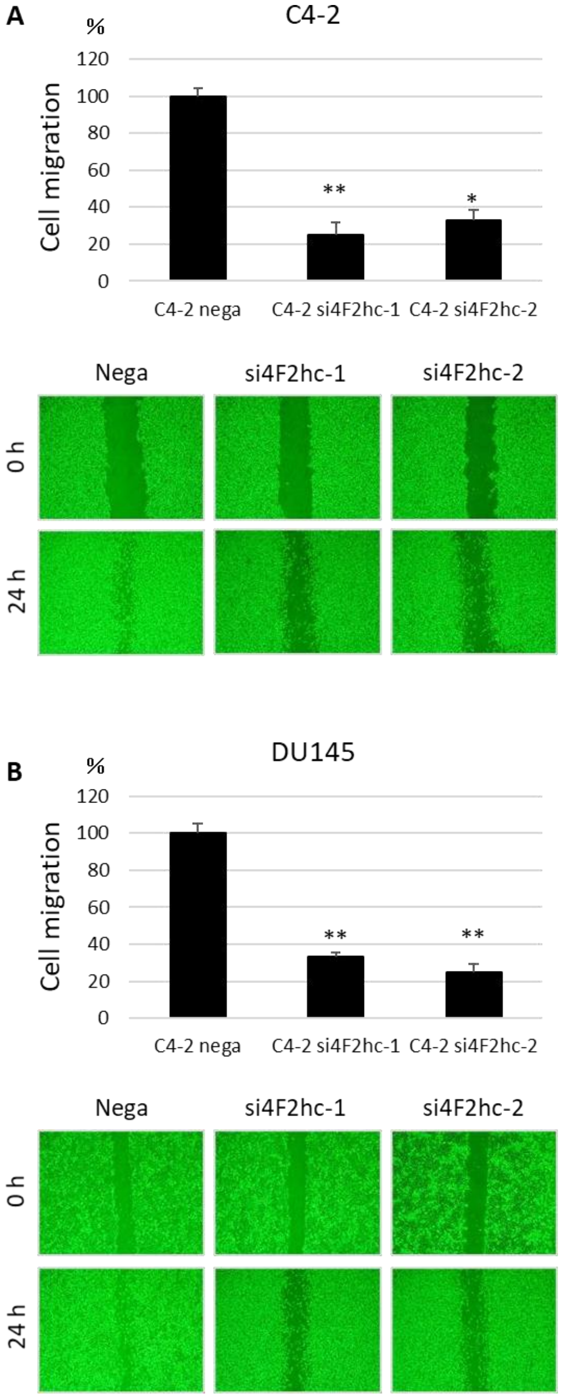

Figure S2. Si4F2hc concentration-dependent effect on candidate genes was assessed by real-time PCR (A). SiSKP2 (siSKP2-1 and siSKP2-2) inhibits DU145 cell proliferation (B). Data represent three independent experiments with similar results. P-values were calculated by the Mann–Whitney U-test. **P<0.01.

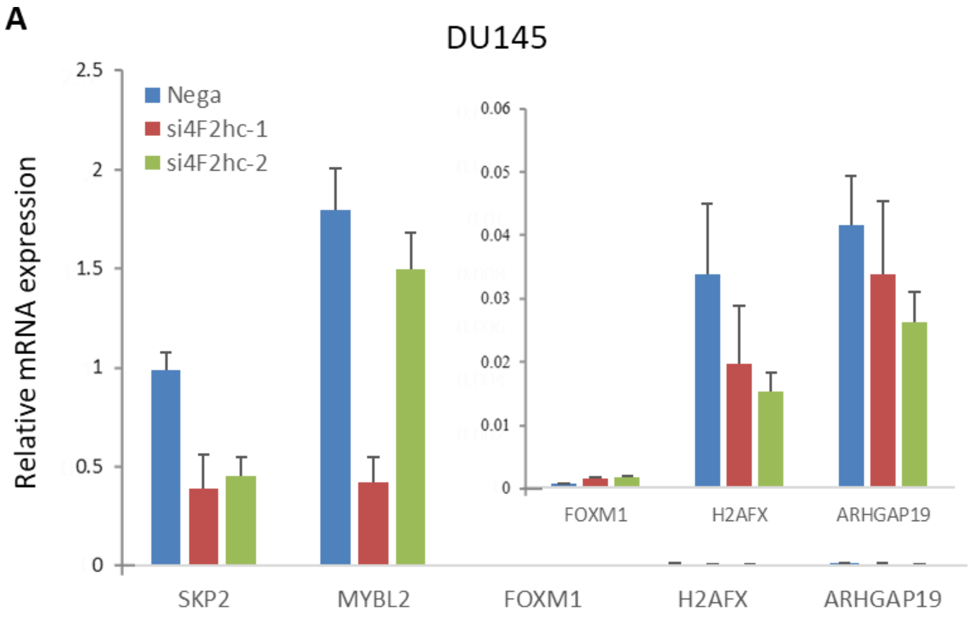

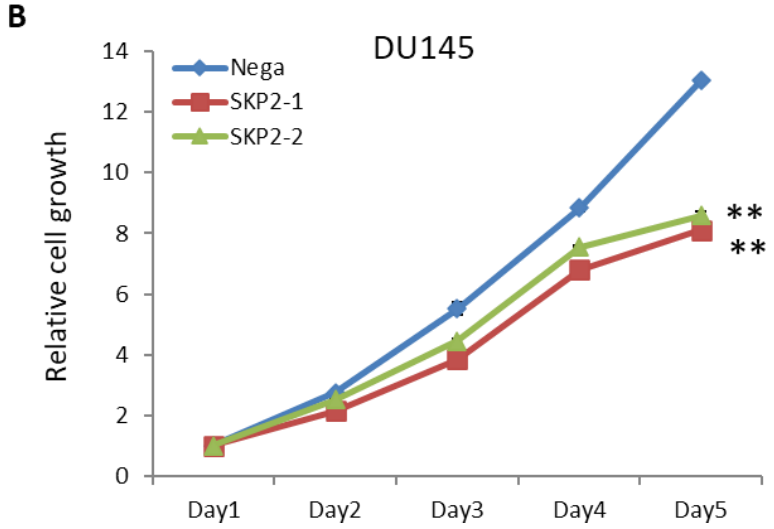

Figure S3. Associations among 4F2hc, SKP, and LAT1 expressions. The expression of 4F2hc in C4-2 cells is inhibited by si4F2hc (A). Knocked down expression of 4F2hc inhibits expression of SKP2 and LAT1 (B and C). The expression of SKP2 in C4-2 cells is inhibited by siSKP2 (D). Knocked down expression of SKP2 in C4-2 cells using siISKP2 does not affect the expression of 4F2hc and LAT1 in mRNA levels (E and F). Nega indicates negative siRNA control. Data represent three independent experiments with similar results. P-values were calculated by the Mann–Whitney U-test. N.S., no significant difference. *P<0.05, **P<0.01.

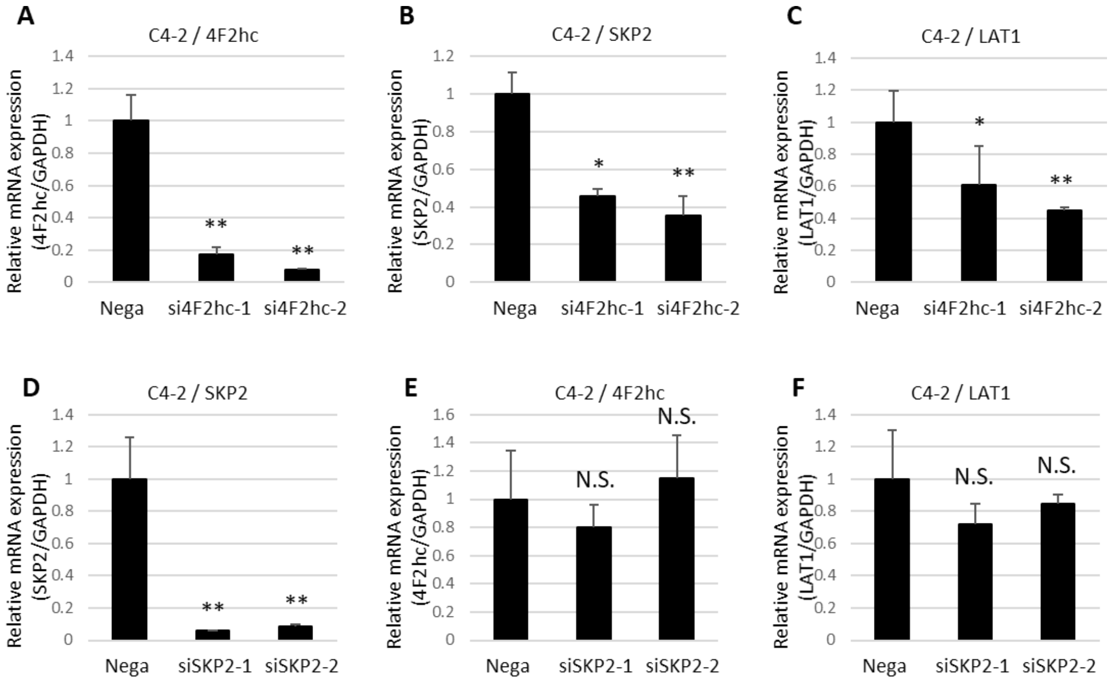

Figure S4. Validation of the association between 4F2hc and SKP2 expression by rescue assay. Inhibition of 4F2hc expression and SKP2 overexpression using C4-2 cells. 4F2hc expression (A), and SKP2 expression (B). SKP2 expression and 4F2hc overexpression were inhibited using C4-2 cells. 4F2hc expression (C), and SKP2 expression (D). Inhibition of 4F2hc expression and SKP2 overexpression in cell proliferation (E). Inhibition of SKP2 expression and 4F2hc overexpression in cell proliferation (F). N.S. No significant difference. **p<0.01, ***p<0.001.

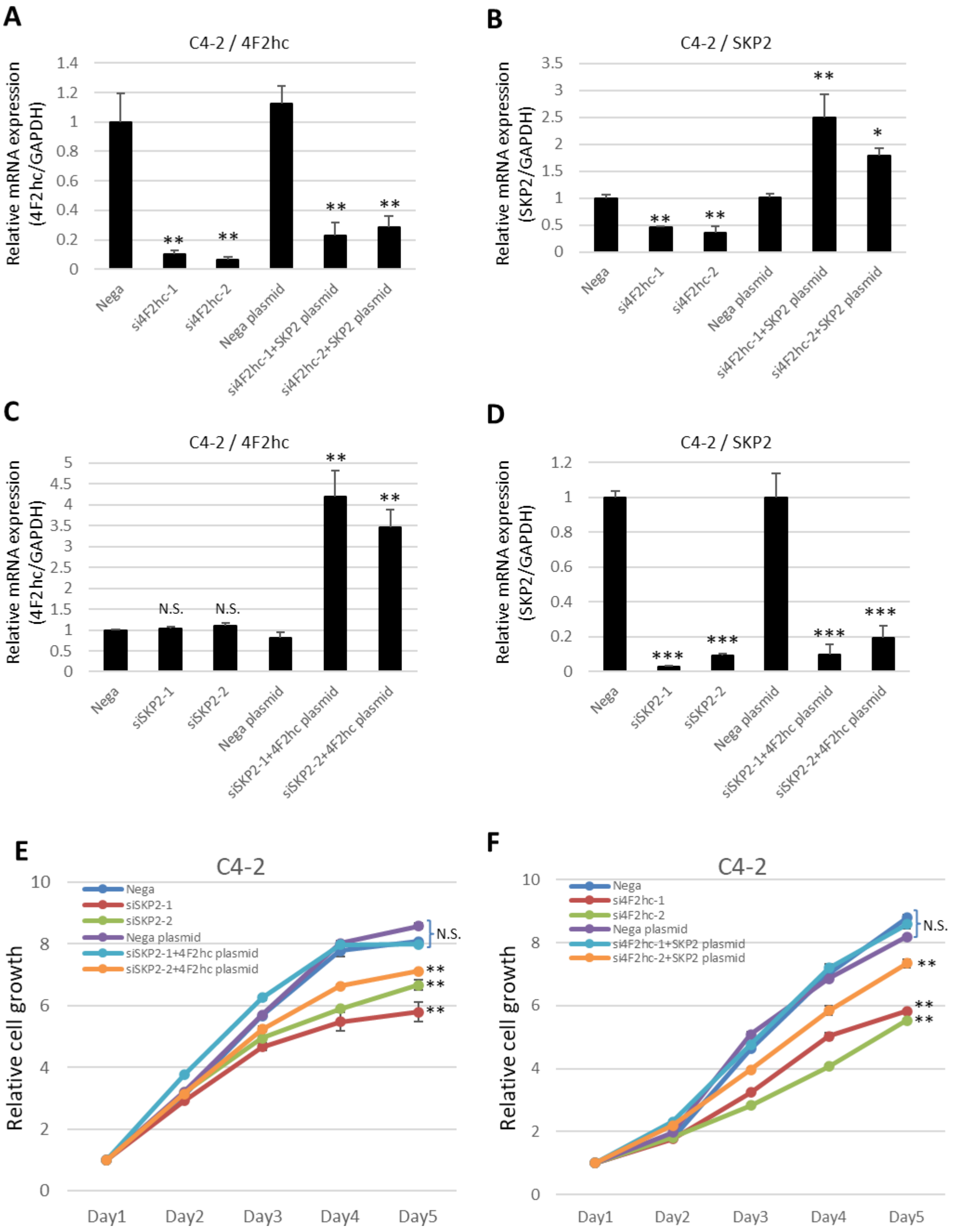

Figure S5. Representative histological sections from prostate cancer tissue. LAT1 expression in PC tissues was analysed by immunohistochemistry. Sections were stained with haematoxylin and eosin (A, a: 600 µm and b: 200 µm). Representative images of LAT1 immunohistochemical expression (c: 600 µm and d: 200 µm).

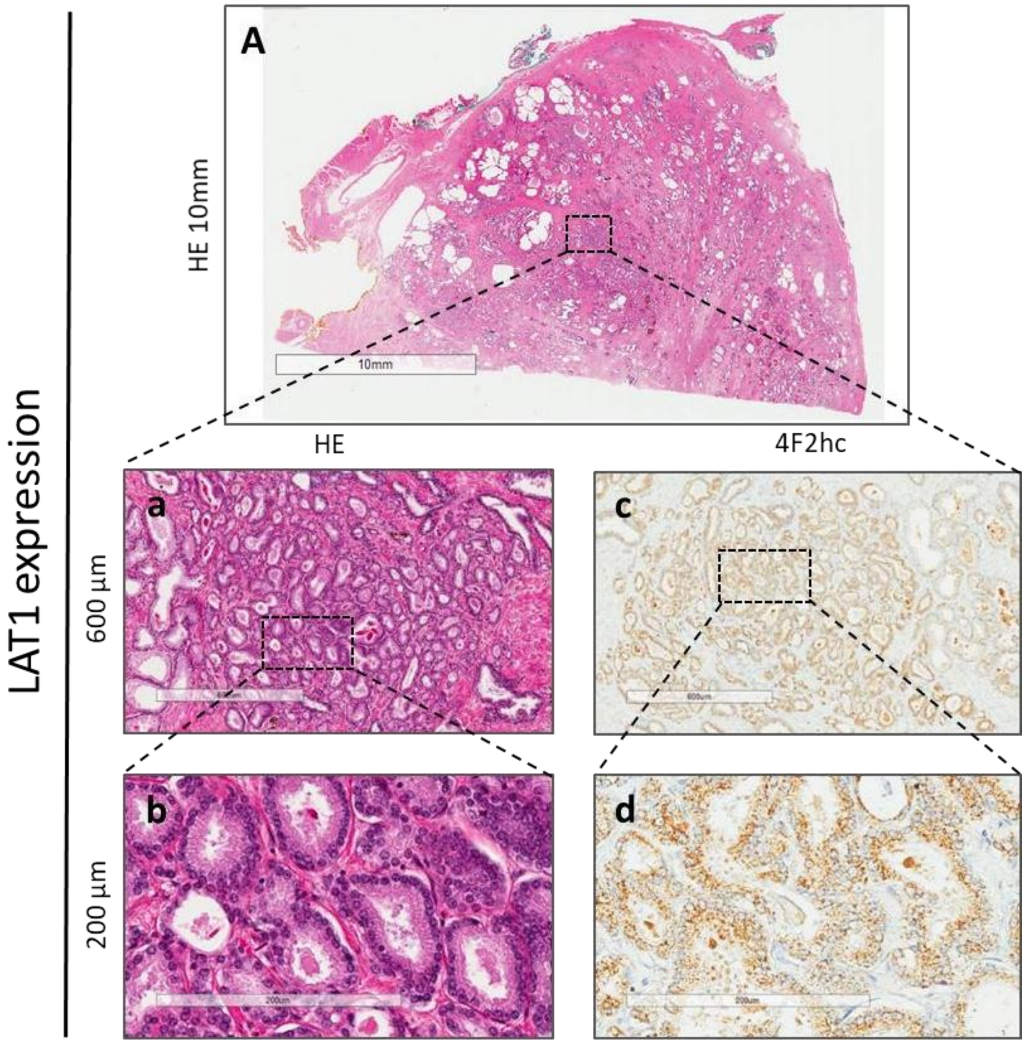

Figure S6. Progression-free survival of PC patients categorized by 4F2hc and LAT1 expression. Prognostic significance of LAT1 expression for PFS (A). Prognostic significance of high 4F2hc/high LAT1 expression and low 4F2hc/lowLAT1 expression. Others are low 4F2hc/high LAT1 or high 4F2hc/low LAT1 (C). N.S. No significant difference. **p<0.01.

A

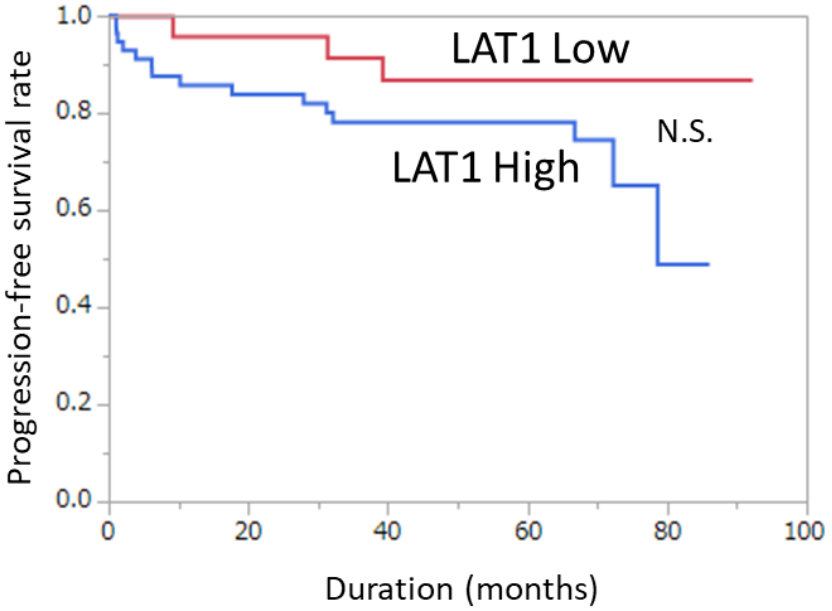

B

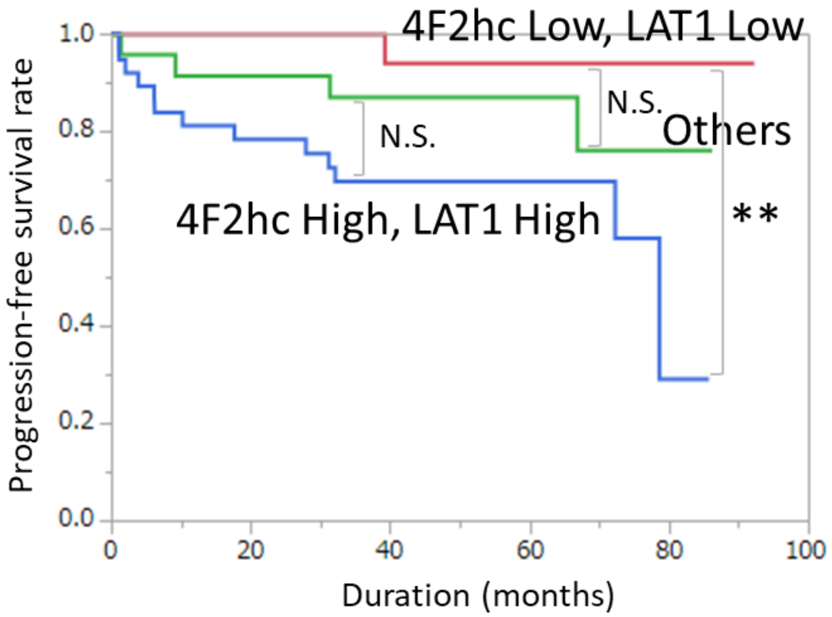

Figure S7. Statistical analysis was performed using the Cancer Genome Atlas (TCGA) data. Prognostic significance of 4F2hc expression for progression-free survival (PFS) (A), and prognostic significance of SKP2 expression for (PFS) (B). Prognostic significance of high 4F2hc/high SKP2 expression and low 4F2hc/low SKP2 expression, others are low 4F2hc/high SKP2 or high 4F2hc/low SKP2 (C). N.S. No significant difference. *P<0.05, **p<0.01.

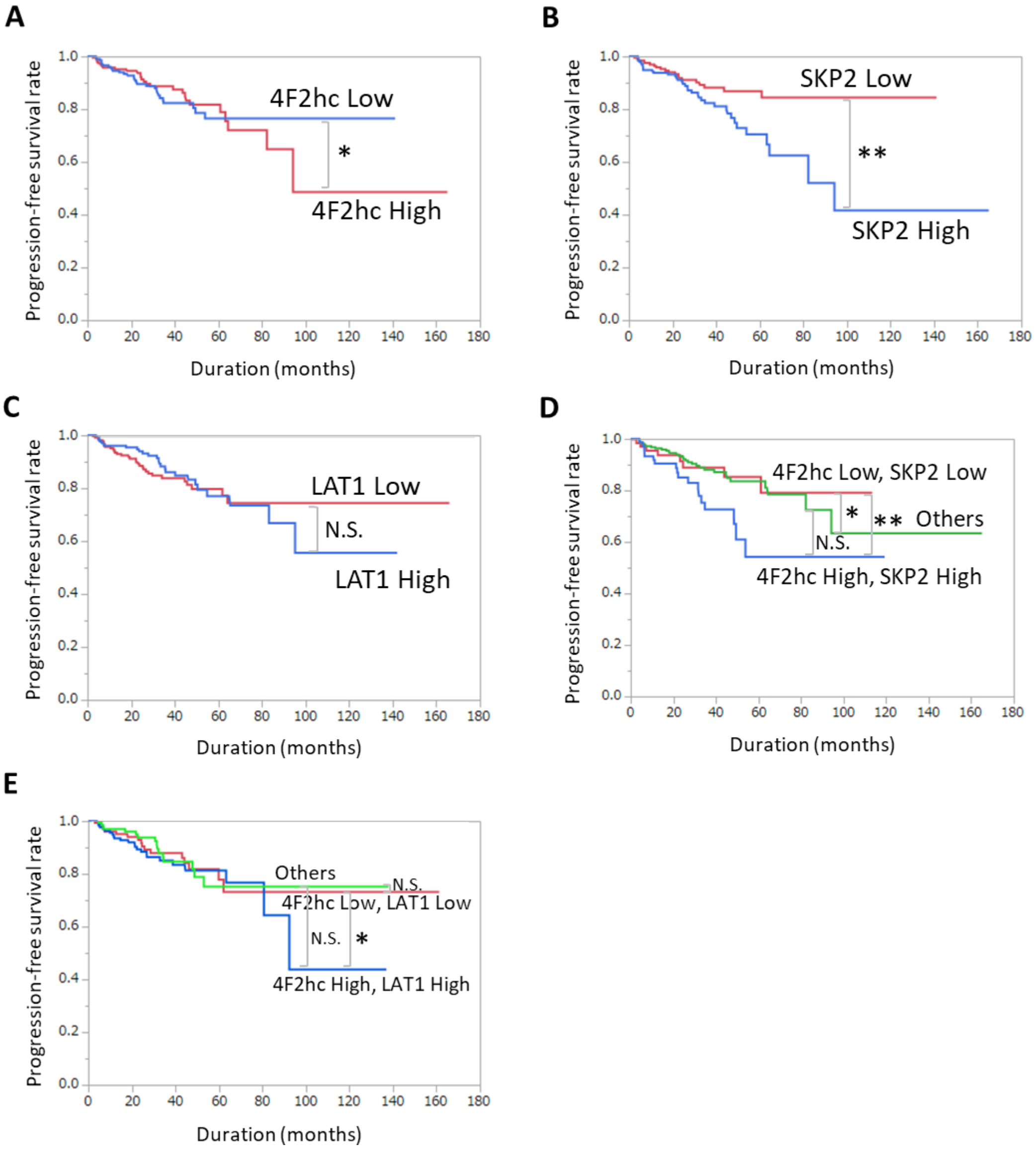

Uncropped Data

Figure1A

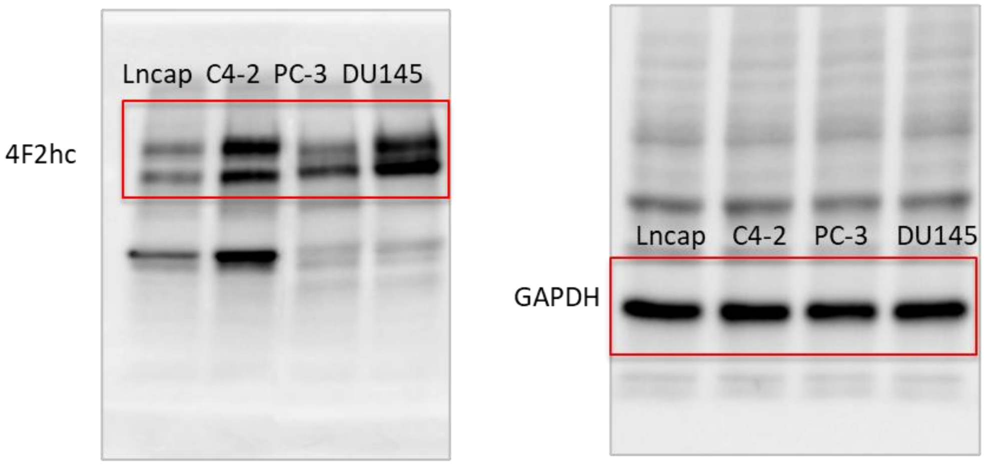

Figure1C

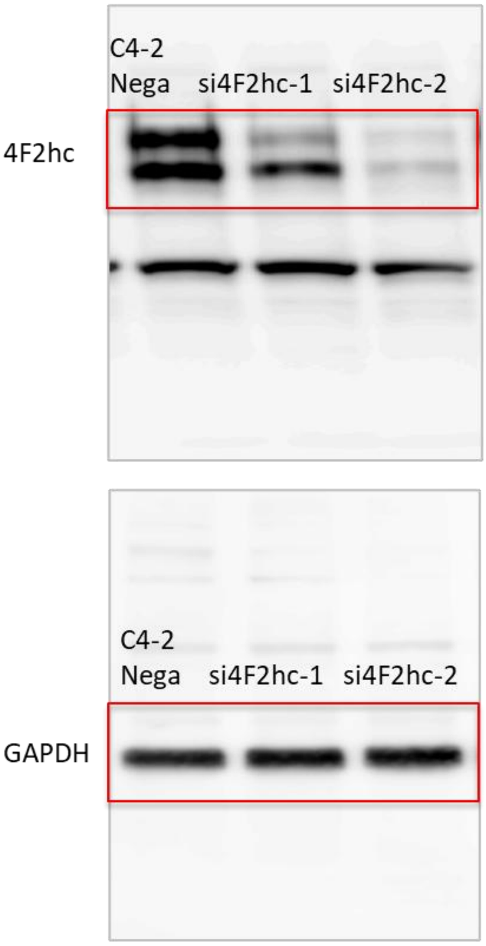

Figure1D

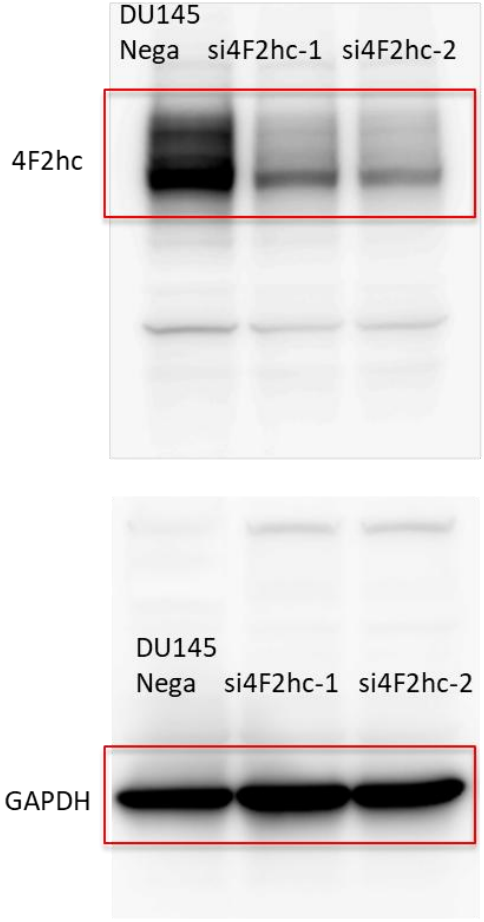

Figure4E

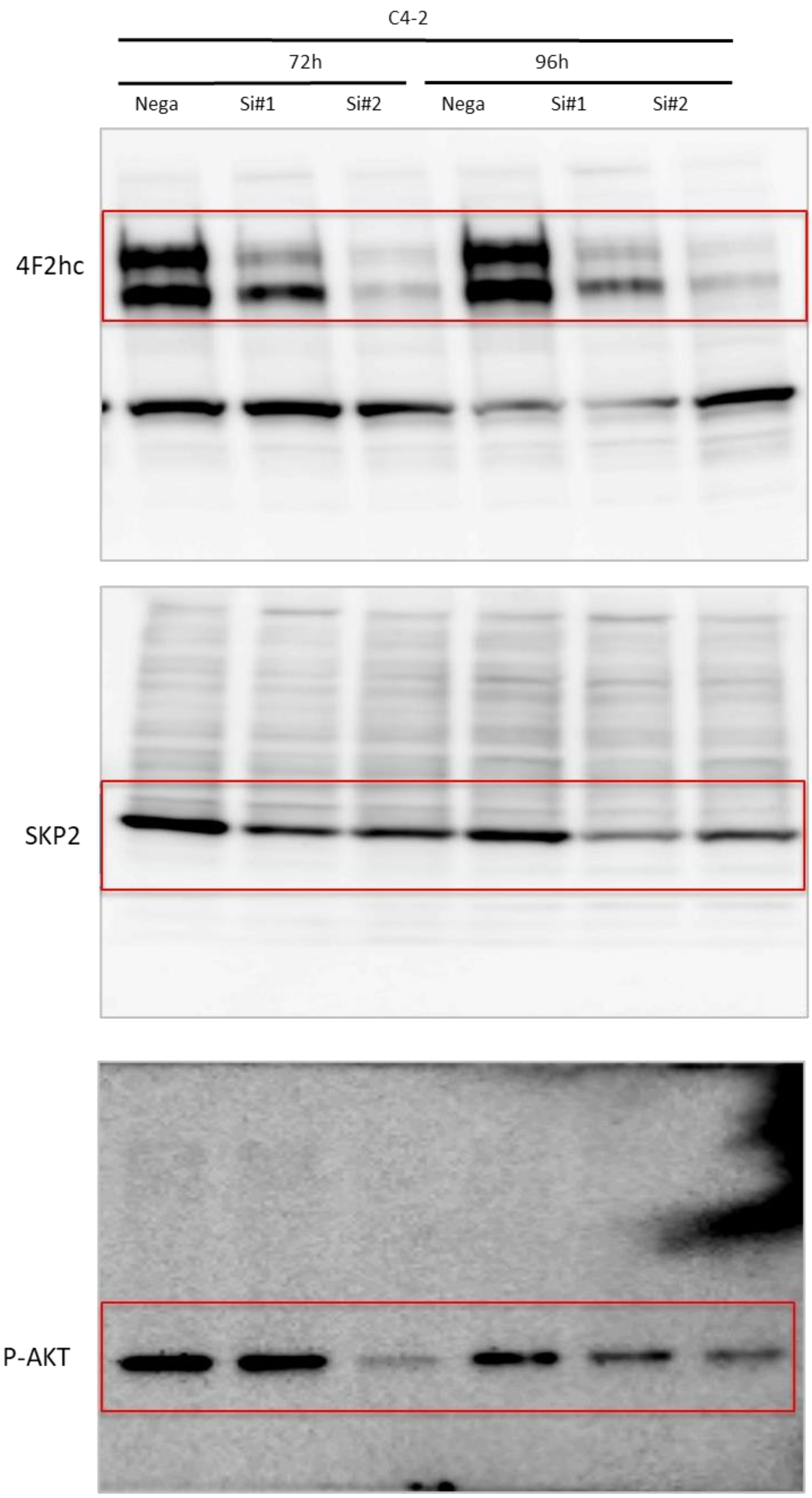

Figure4E

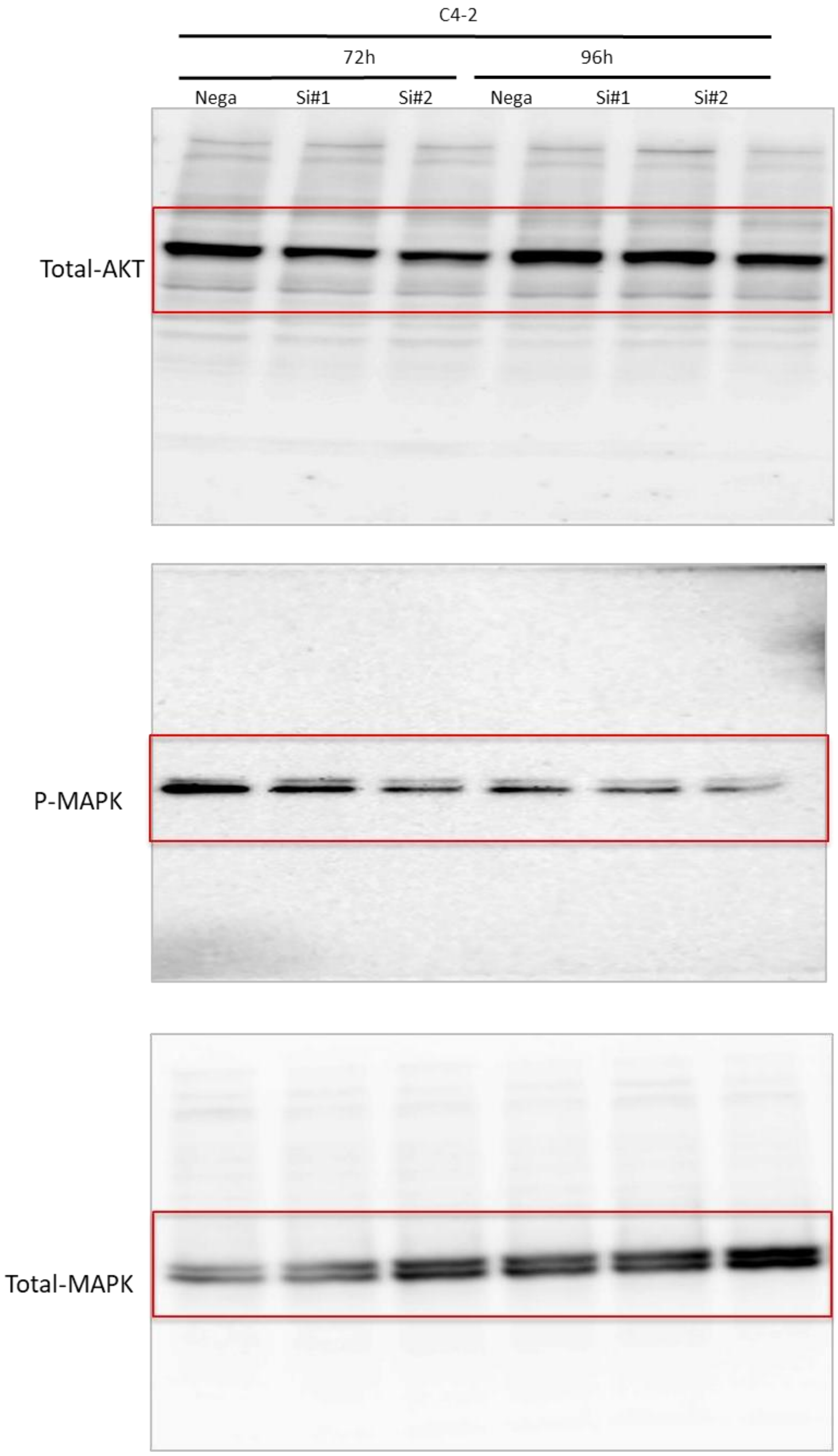
C4-2
72h
96h
Nega
Si#1
Si#2
Nega
Si#1
Si#2
Total-AKT
P-MAPK
Total-MAPK

Figure4E

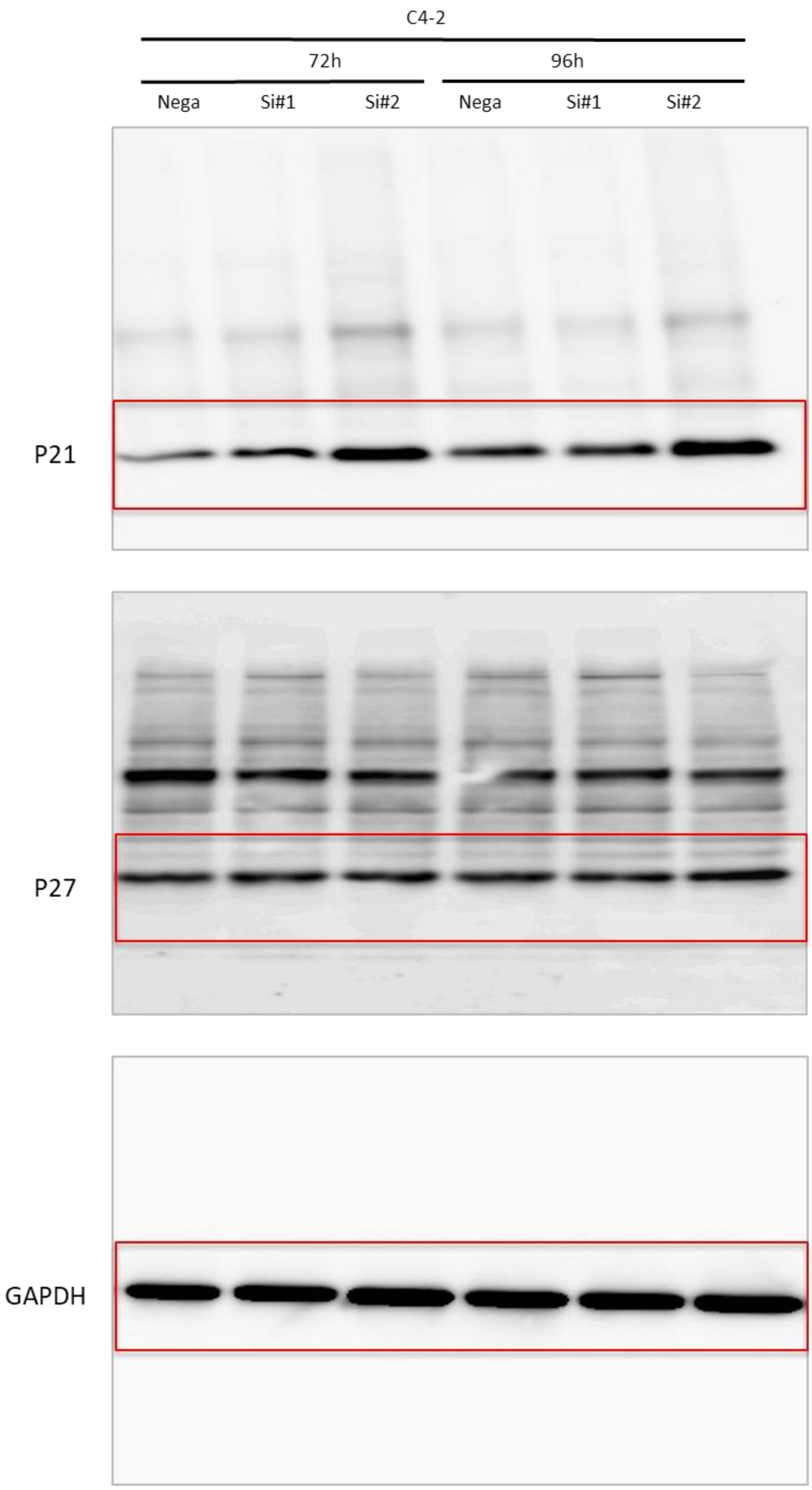

Supplement: Supplementary file 1 — Supplementary Information. [file 41598_2021_90748_MOESM1_ESM.pdf]
